# Supplementary material for: Anti-Inflammatory and Gut Microbiota Modulation Potentials of Flavonoids Extracted from Passiflora foetida Fruits
Source: Foods. 2023 Jul 29;12(15):2889. doi: 10.3390/foods12152889 (PMC10417441; doi:10.3390/foods12152889)
Supplement: Supplementary file 1 [file foods-12-02889-s001.zip › foods-2520871-supplementary.pdf]

Anti-Inflammatory and Gut Microbiota Modulation Potentials of Flavonoids Extracted from *Passiflora foetida* Fruits

Xiangpeng Han [1,2,†], Ya Song [1,2,†], Riming Huang [1,2], Minqian Zhu [1,2], Meiying Li [1,2], Teresa Requena [3,*] and Hong Wang [1,2,*]

[1] Guangdong Provincial Key Laboratory of Food Quality and Safety, College of Food Science, South China Agricultural University, Guangzhou 510642, China; hanxp1996@163.com (X.H.); songya_1990@163.com (Y.S.); huangriming@scau.edu.cn (R.H.); zhuminqian@stu.scau.edu.cn (M.Z.); lmy1982@scau.edu.cn (M.L.)
[2] Guangdong Laboratory for Lingnan Mordern Agriculture, Guangzhou 510642, China
[3] Instituto de Investigación en Ciencias de la Alimentación CIAL (CSIC), Campus UAM Cantoblanco, 28049 Madrid, Spain
* Correspondence: t.requena@csic.es (T.R.); gzwhongd@scau.edu.cn (H.W.)
† These authors contributed equally to this work.

Citation: Han, X.; Song, Y.; Huang, R.; Zhu, M.; Li, M.; Requena, T.; Wang, H. Anti-Inflammatory and Gut Microbiota Modulation Potentials of Flavonoids Extracted from *Passiflora foetida* Fruits. *Foods* **2023**, *12*, x. https://doi.org/10.3390/xxxxx

Academic Editor: Victor Rodov

Received: 7 July 2023
Revised: 25 July 2023
Accepted: 25 July 2023
Published: 27 July 2023

Copyright: © 2023 by the authors. Submitted for possible open access publication under the terms and conditions of the Creative Commons Attribution (CC BY) license (https://creativecommons.org/licenses/by/4.0/).

Table S1 Primers and amplification conditions for real-time PCR assays.

Bacterial group	Primers	Primer sequence and direction (5′–3′)	Product size (bp)	Annealing T (°C)	Time (s)
Alistipes	AlstpF<br>AlstpR	F: 5′ GTACTAATTCCCCATAACATTCGAG 3′<br>R: 5′ CTAATACAACGCATGCCCATCTT 3′	83	60	40
Bifidobacterium	Bif-F<br>Bif-R	F: 5′ CTCCTGGAAACGGGTGG 3′<br>R: 5′ GGTGTTCTTCCCGATATCTACA 3′	593	55	40
Bilophia	BwF<br>BwR	F: 5′ AAGTCCTTCGGGGCGAGTAA 3′<br>R: 5′ ATCCTCTCAGACCGGCTAC 3′	239	60	40
Enterobacteriaceae	F-ent<br>R-ent	F: 5′ ATGGCTGTCGTCAGCTCGT 3′<br>R: 5′ CCTACTTCTTTTGCAACCCACTC3′	385	55	40
Enterococcus	g-Encoc-F<br>g-Encoc-R	F: 5′ ATCAGAGGGGGATAACACTT 3′<br>R: 5′ ACTCTCATCCTTGTTCTTCTC 3′	336	55	40
Lactobacillus	Lab-F362<br>Lab-R677	F: 5′ AGCAGTAGGGAATCTTCCA 3′<br>R: 5′ CACCGCTACACATGGAG 3′	341	58	40
Roseburia	RosF1<br>RosR1	F: 5′ GCGGTRCGGCAAGTCTGA 3′<br>R: 5′ CCTCCGACACTCTAGTMCGAC 3′	81	60	40

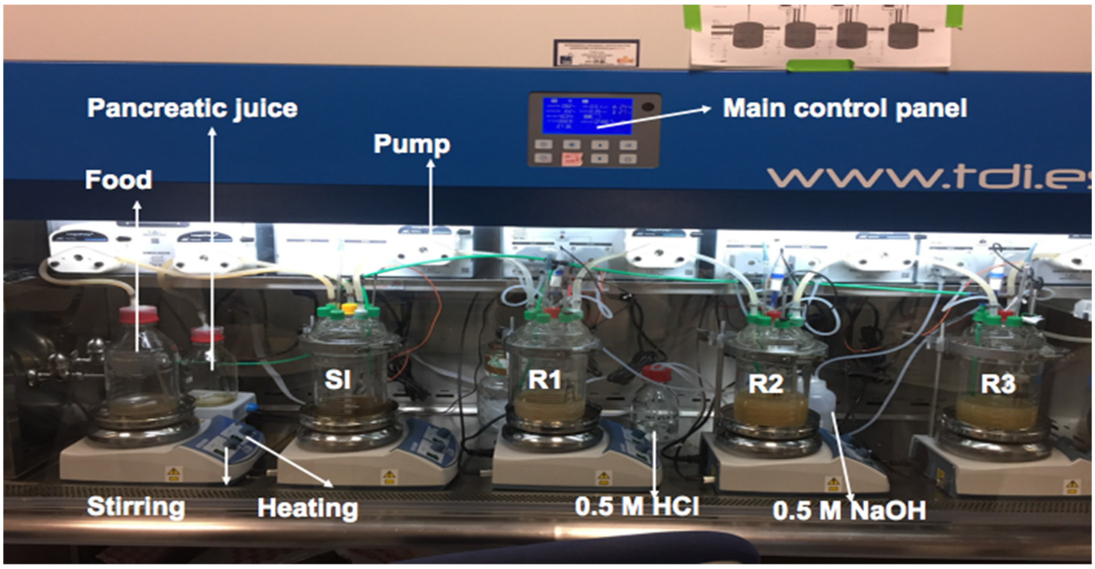

Figure S1 Schematic diagram of the BFBL gut model.

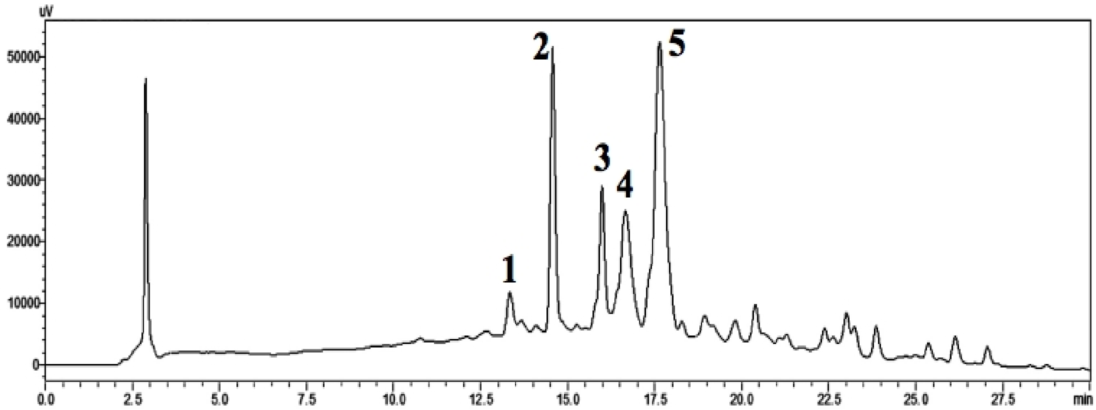

Figure S2 HPLC chromatography spectrum of PFF.

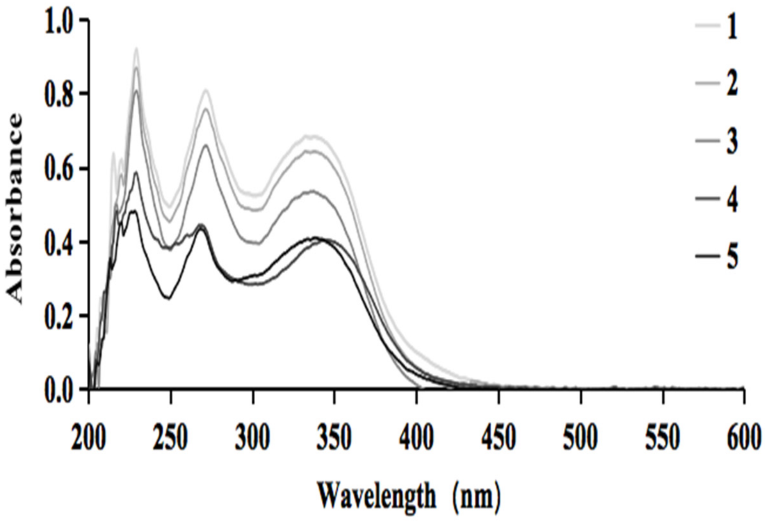

Figure S3 The UV spectra of purified compounds **1-5**.

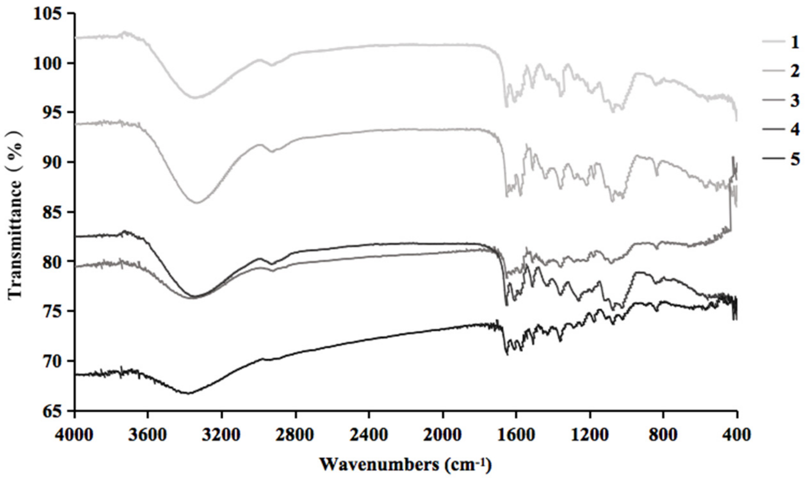

Figure S4 The FT-IR spectra of purified compounds **1-5**.

A

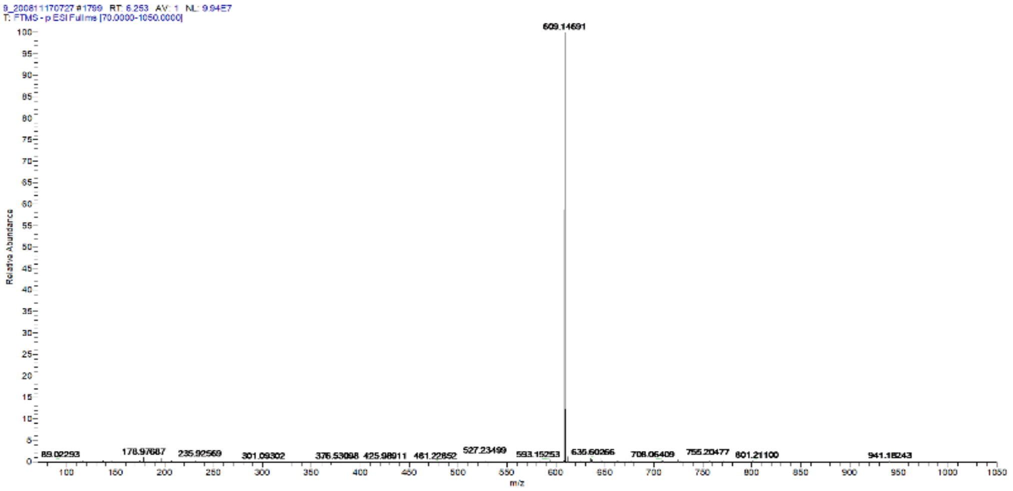

B

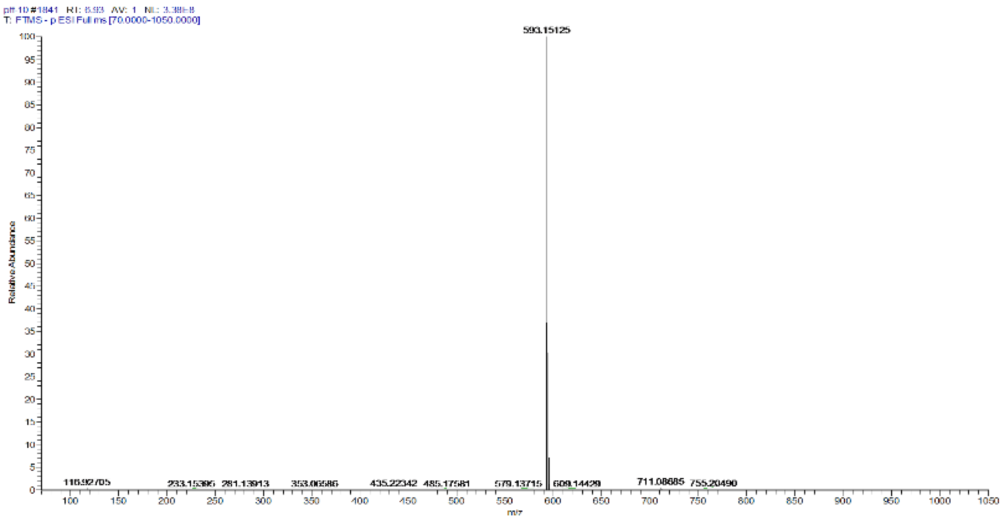

C

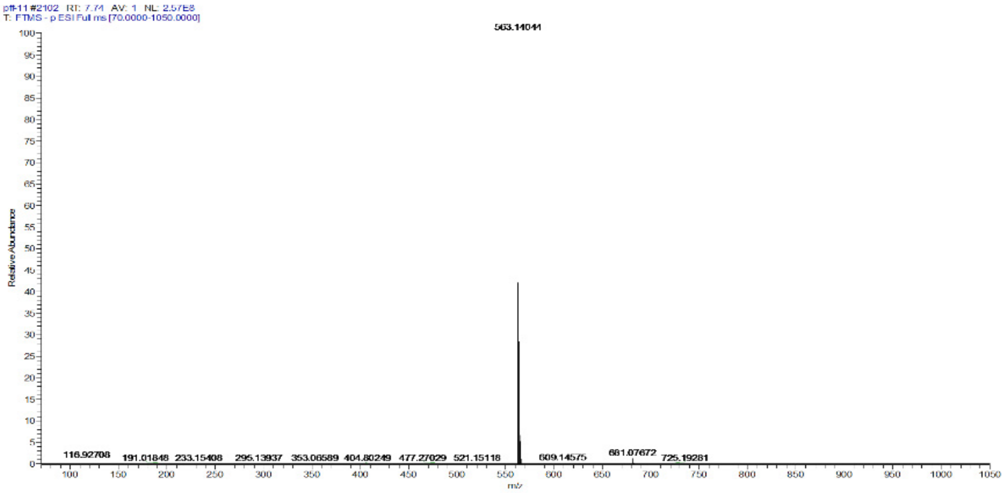

D

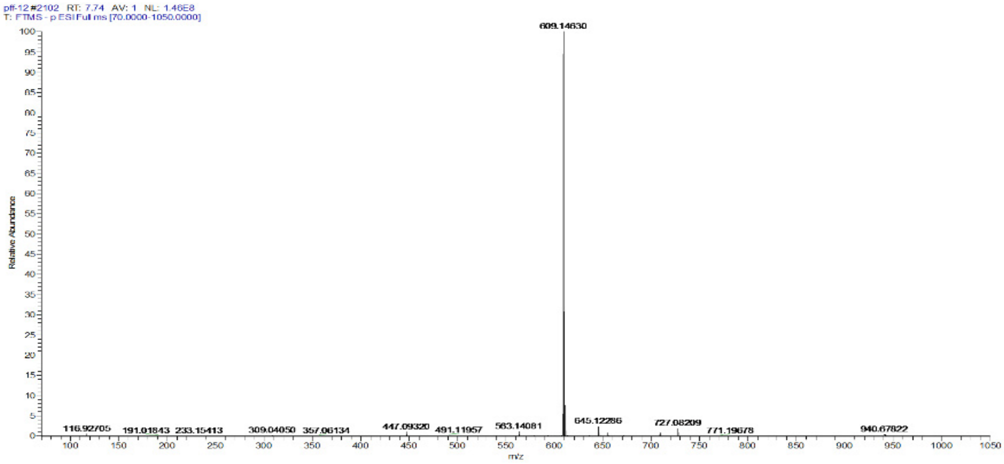

E

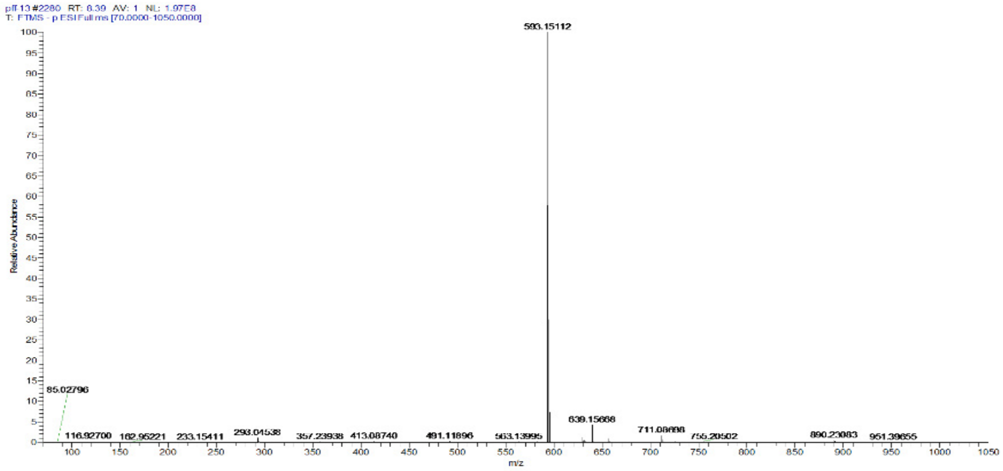

Figure S5 The LC-ESI-MS spectra of purified compound **1** (A), **2** (B), **3** (C), **4** (D), and **5** (E).
